# U-Shaped Association of Aspect Ratio and Single Intracranial Aneurysm Rupture in Chinese Patients: A Cross-Sectional Study

**DOI:** 10.3389/fneur.2021.731129

**Published:** 2021-11-03

**Authors:** Jia-He Yin, Shi-Xing Su, Xin Zhang, Yi-Ming Bi, Chuan-Zhi Duan, Wei-mei Huang, Xi-Long Wang

**Affiliations:** ^1^National Key Clinical Specialty/Engineering Technology Research Center of Education Ministry of China, Guangdong Provincial Key Laboratory on Brain Function Repair and Regeneration, Neurosurgery Institute, Department of Neurosurgery, Zhujiang Hospital, Southern Medical University, Guangzhou, China; ^2^Department of Interventional Treatment, Southern Medical University, Guangzhou, China; ^3^Department of Traditional Chinese Medicine, Southern Medical University, Guangzhou, China

**Keywords:** aspect ratio (AR), rupture of intracranial aneurysm, association, generalized additive model, subgroup analysis

## Abstract

**Background:** Previous studies have analyzed the association of aspect ratio (AR) on the ruptured intracranial aneurysm (IA), but the findings are inconclusive and controversial. Therefore, the study aimed to derive a more detailed estimation of this association between AR and ruptured IA in Chinese IA patients.

**Methods:** The present work was a cross-sectional study. We retrospectively collected 1,588 Chinese patients with a single IA from January 2010 to November 2017. The relationship was examined between AR at diagnosis and ruptured IA. Covariates included data of demographics, morphological parameters, lifestyle habits, clinical features, and comorbidities. Binary logistic regression and two-piecewise linear models were used to analyze independent associations of AR with ruptured IA.

**Results:** The results suggest that the association between AR and IA rupture was U-shaped. In the AR range of 1.08–1.99, the prevalence of IA rupture was 13% lower for each 0.1-unit increment in AR [odds ratio 0.87, 95% confidence interval (CI) 0.80–0.98]. Conversely, for every 0.1-unit increase in AR, the prevalence of IA rupture increased by ~3% (odds ratio 1.03, 95% CI 1.01–1.06) in the AR range of 3.42–4.08.

**Conclusion:** The relationship between AR and ruptured IA was U-shaped, with the negative association at AR of 1.08–1.99 and positive association at AR of 3.42–4.08.

## Introduction

Due to methodological limitations, the exact prevalence of intracranial aneurysms (IA) and the risk of rupture remains unclear (1). A current review suggests that guidelines (the American Heart Association/American Stroke Association (AHA/ASA) guideline) underestimate the incidence of aneurysm rupture (1). This review suggested that at least 1 in 20–30 adults is likely to carry an unruptured IA and approximately one-quarter of these UIAs rupture in a lifetime (1). In China, the prevalence of IA is roughly the same as in Western countries, but the exact incidence of IA rupture has not been reported (2). Based on China's huge population base, therefore, establishing an algorithm to stratify the risk of IA rupture is needed.

The prerequisites of predicted model development are based on the full clarification of ruptured IA-associated factors (3). Although the discovery of IA rupture-associated factors is still underway, it is widely accepted that the morphological parameters of IA are closely related to rupture. As an essential and the most used morphological indicator, aspect ratio (AR) was therefore incorporated as a candidate for constructing a model in most of these model-driven studies or IA rupture-associated factors discovery (4–8). However, a review reported that existing studies devoted to determining the correlations between IA rupture and AR yield mixed results (7). In addition, most studies have a small sample size, and rarely discuss the non-linear relationship and the interaction among AR and other morphological parameters. Because non-linear relationships have a significant impact on the evaluation of effect size or model development (3, 9, 10), we, therefore, present a large-scale cross-sectional study to investigate the association between AR and rupture risk of Chinese patients with single IA.

## Participants and Methods

### Study Design

We performed a cross-sectional study based on a large sample. The interesting independent variable is AR. The outcome variable is the rupture status of a single IA (dichotomous variable: 1 = rupture; 0 = unrupture).

### Study Population

There were 1,786 patients with IA initially collected between January 2010 and November 2017 at the Department of Neurosurgery, Zhujiang Hospital. Diagnosis of IA for all involved patients was confirmed by magnetic resonance angiography (MRA) or computed tomography angiography (CTA) examination or by digital subtraction angiography (DSA) after admission. We excluded individuals with multiple IA (*n* = 55), feeding artery aneurysms-associated arteriovenous malformations (*n* = 67), fusiform IA (*n* = 29), or dissecting aneurysms (*n* = 47), leaving 1,588 individuals for the main analysis. Clinical information for each IA patient was obtained from our electronic medical record system. Participants' informed consent is not required in this study because of the nature of the retrospective study. The hospital institutional review board approved this study.

### The Measurement of Morphological Parameters

All 1,588 IAs were examined by digital subtraction angiography (DSA). The patient-specific geometries were reconstructed from 3D-DSA images to confirm the morphological parameters (11, 12).

*Aspect ratio*: The aneurysm AR was determined as the ratio of the greatest perpendicular height to the mean neck diameter, where the mean neck diameter was evaluated as twice the mean distance from the neck centroid to the edge of the neck (12).

*Size ratio*: The aneurysm-to-vessel size ratio (SR) integrates the geometries of the IA and its parent vessel and was explained as SR = (maximum aneurysm height)/(average vessel diameter) (12).

*IA size*: IA size was defined as the largest perpendicular distance of the dome from the neck plane (12).

### Outcome Variable

The outcome in this study was IA rupture. The diagnosis of subarachnoid hemorrhage was confirmed by computed tomography (CT) scan.

### Other Involved Covariates

We collected information on covariates that are known risk factors for IA (13–22). Consequently, the following variables were included: age (year), cerebral microbleeds (yes, no), IA position (PcoA: posterior communicating artery; AcoA: anterior communicating artery; ICA: internal carotid artery; ACA: anterior cerebral artery; MCA: middle cerebral artery; VA: vertebrobasilar artery), sex (male, female), hypertension history (yes, no), diabetes history (yes, no), atherosclerosis (yes, no), hyperlipidemia (yes, no), coronary artery disease (CAD, yes, no), current smoker (yes, no), current alcohol consumption (yes, no), Willis variation (yes, no), the shape of IA (henceforth shape; regular or irregular), and neck status of IA (henceforth neck; wide or narrow). The history of comorbidities was self-reported by patients or blood relatives.

### Missing Data Addressing

The proportion of missing data was low (<5%) for all variables, multiple imputation was not used.

### Statistical Analysis

Continuous variables are presented as mean (SD) and categorical variables as No. (%). Differences between the unruptured and ruptured groups were evaluated using chi-square test for categorical variables and using *t* test for continuous variables. Multivariable logistic regression was performed to determine the association between AR and ruptured IA. We present both univariate and multivariate models. In the multivariate model, we included those variables that showed statistical differences between the two groups.

We also test the possibility of a non-linear relation of AR with ruptured IA. By the recurrence method, we calculated the inflection points of the U-shaped curve. Afterward, by the two-piecewise linear regression model, we divided the U-shaped curve according to the inflection point, and established the two-piecewise linear model on both sides of the inflection points, and calculated the OR and confidence intervals, respectively.

All the analyses were performed with the statistical software packages R (http://www.R-project.org, The R Foundation) and EmpowerStats (http://www.empowerstats.com, X&Y Solutions, Inc, Boston, MA). *P* < 0.05 (two-sided) were considered statistically significant.

## Results

### Baseline Characteristics of Selected Participants

The overall prevalence of ruptured IA was 41.75% (*n* = 663). At baseline, the mean age of participants was 59.69 years, and the proportion of men was 40.0%. Table 1 describes the baseline characteristics of the patients. There were no statistically significant differences between non-ruptured and ruptured groups in terms of angle, diabetes history, atherosclerosis history, current alcohol consumption, or Willis variation (all *P* > 0.05). The ruptured group contained more patients who were older and had larger IA size, mean neck diameter, and comorbidity, more likely to be located in AcoA, PcoA, or MCA compared with patients with unruptured IA. In terms of morphological parameters, those in the ruptured group had lower SR, with a higher proportion of irregular shape, wide neck, and CMBs compared with the unruptured group.

**Table 1:** The baseline characteristics of patients with intracranial aneurysm.

**Variables**	**Unruptured IA**	**Ruptured IA**	***P***
***N***	925	663	
**Age, mean (SD), year**	59.18 (10.95)	60.58 (10.81)	0.010
**Intracranial aneurysm size, mean (SD), mm**	7.06 (4.26)	7.57 (4.00)	0.012
**AR, mean (SD)**	2.47 (0.69)	2.38 (0.77)	0.008
**Aneurysm angle, mean (SD), degree**	133.01 (28.72)	134.13 (29.34)	0.274
**Size ratio**	2.82 (1.20)	2.76 (1.36)	0.019
**Mean neck diameter, mean (SD), mm**	3.17 (2.50)	3.67 (2.72)	<0.001
**Aneurysm location**			<0.001
AcoA	207 (22.38%)	211 (31.83%)	
ICA	225 (24.32%)	53 (7.99%)	
ACA	67 (7.24%)	42 (6.33%)	
VA	134 (14.49%)	7 (1.06%)	
PcoA	213 (23.03%)	208 (31.37%)	
MCA	79 (8.54%)	142 (21.42%)	
**Sex**			0.005
Male	380 (41.08%)	242 (36.50%)	
Female	545 (58.92%)	421 (63.50%)	
**Hypertension history (self-report)**			<0.001
No	620 (67.89%)	351 (52.94%)	
Yes	291 (32.11%)	312 (47.06%)	
Missing	14 (1.51%)	9 (1.36%)	
**Diabetes (self-report)**			0.188
No	788 (85.19%)	571 (86.12%)	
Yes	106 (11.46%)	62 (9.35%)	
Missing	31(3.35%)	30 (4.53%)	
**Atherosclerosis**			0.986
No	773 (85.51%)	553 (83.41%)	
Yes	104 (14.49%)	90 (13.57%)	
Missing	48 (5.19%)	20 (3.02)	
**Hyperlipidemia**			0.003
No	623 (67.35%)	489 (73.76%)	
Yes	266 (28.76%)	149 (22.47%)	
Missing	36 (3.89%)	25 (3.77%)	
**Coronary artery disease (self-report)**			<0.001
No	786 (84.97%)	588 (88.69%)	
Yes	101 (10.92%)	48(7.24%)	
Missing	38 (4.11%)	27(4.07%)	
Current smoker (self-report)			0.009
No	788 (85.19%)	592 (89.36%)	
Yes	137 (14.81%)	71 (10.64%)	
Current alcohol consumption (self-report)			0.375
No	776 (83.89%)	546 (82.36%)	
Yes	149 (16.11%)	117 (17.64%)	
Willis variation			0.890
No	251 (27.14%)	178 (26.85%)	
Yes	674 (72.86%)	485 (73.15%)	
Shape			<0.001
Regular	752 (81.30%)	384 (57.85%)	
Irregular	173 (18.70%)	279 (42.15%)	
Neck			<0.001
Wide	181 (19.57%)	174 (26.2%)	
Narrow	744 (80.43%)	489 (73.8%)	
CMBs			<0.001
No	863 (94.30%)	594 (89.62%)	
Yes	62 (5.70%)	69 (10.38%)	

*CMBs, cerebral microbleeds; AR, aspect ratio; PcoA, The posterior communicating artery; AcoA, anterior communicating artery; ICA, internal carotid artery; ACA, anterior cerebral artery; MCA, middle cerebral artery; VA, vertebrobasilar artery; FBS, fasting blood sugar*.

### Results of Multivariable Logistic Regression

We put the variables with significant differences between the two groups in Table 1 into univariate and multivariate models (Table 2). The unadjusted OR for continuous AR (per 0.1-unit increase) and prevalence of ruptured IA was 0.98 (95% CI 0.97–1.00). This result means every 0.1 unit increase in AR was associated with 2% lower prevalence of ruptured IA. After adjusting for covariates presented in Table 1, a linear association of AR with ruptured IA was not observed (adjusted-OR: 1.19, 95% CI 1.04–1.35).

**Table 2:** Univariable and multivariable analysis by binary logistic regression.

**Variables**	**Univariable**	**Multivariable**
	**OR, 95% CI**	**OR, 95% CI**
**Age**	1.01 (1.00, 1.02)	1.01 (1.00, 1.03)
**IA size**	1.03 (1.01, 1.05)	1.04 (1.01, 1.07)
**Aspect ratio(per 0.1 change)**	0.98 (0.97, 1.00)	1.00 (0.98, 1.03)
**Size ratio**	1.03 (0.89, 1.05)	1.05 (0.82, 1.98)
**Mean neck diameter**	1.08 (1.04, 1.12)	1.12 (0.78, 1.25)
**IA position**		
AcoA	Reference	Reference
ICA	0.23 (0.16, 0.33)	0.31 (0.21, 1.45)
ACA	0.61 (0.40, 0.95)	0.58 (0.37, 8.91)
VA	0.05 (0.02, 0.11)	0.06 (0.03, 9.14)
PcoA	0.96 (0.73, 1.26)	0.98 (0.72, 1.32)
MCA	1.76 (1.26, 2.47)	2.75 (1.89, 4.00)
**Sex**		
Male	Reference	Reference
Female	1.21 (0.99, 1.49)	1.19 (0.94, 1.52)
**Hypertension history**		
No	Reference	Reference
Yes	1.88 (1.53, 2.31)	1.90 (1.50, 2.41)
**Hyperlipidemia**		
No	Reference	Reference
Yes	0.89 (0.71, 1.12)	1.19 (0.90, 1.56)
**Atherosclerosis**		
No	Reference	Reference
Yes	1.02 (0.77, 1.35)	0.92 (0.67, 1.27)
**Smoking status**		
Non-smoker	Reference	Reference
Current smoker	1.16 (0.88, 1.53)	1.10 (1.05 1.51)
**CMBs**		
No	Reference	Reference
Yes	2.04 (1.36, 3.06)	1.60 (1.04, 2.48)
**Coronary artery disease history**		
No	Reference	Reference
Yes	0.54 (0.38, 0.77)	0.47 (0.31, 1.72)
**Shape**		
Regular	Reference	Reference
Irregular	3.05 (2.44, 3.83)	2.55 (1.97, 3.30)
**Neck**		
Wide	Reference	Reference
Narrow	0.72 (0.57, 0.91)	0.67 (0.46, 1.99)

Results obtained from multivariable logistic regression have indicated that increasing age, higher IA size, patients with a history of hypertension, current smoker, higher size ratio, concurrent cerebral microbleeds, aneurysm located in MCA, and irregular shape IA are associated with a high risk of aneurysm rupture.

In addition, when the multivariate logistic regression model controls for confounding factors, we failed to observe the relationship between gender, history of hyperlipidemia, history of CAD, size ratio, mean neck diameter, and the state of aneurysm neck and rupture of an aneurysm.

### The Results of Non-linearity of AR and Ruptured IA

**Figure 1:**
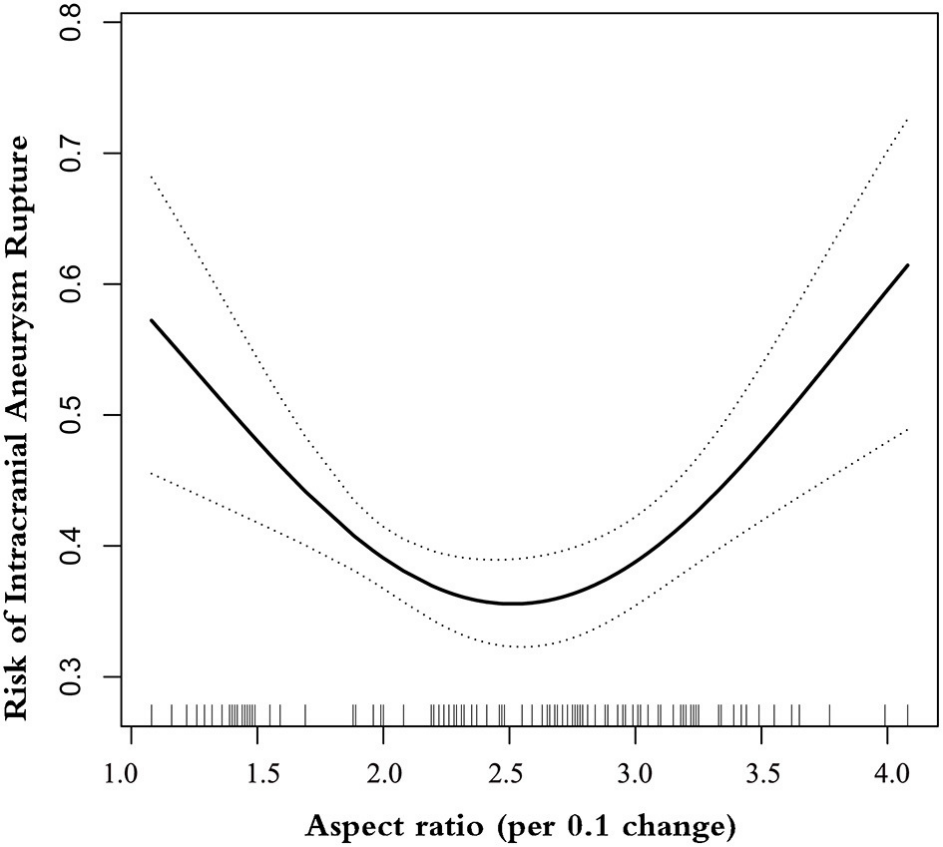
The U-shaped association between AR and risk of ruptured IA.

A smooth curve-fitting analysis observed a U-shaped relationship of AR correlation to IA rupture (Table 3 and Figure 1). By recursive algorithm, we got the inflection points of 1.99 and 3.42. In the AR range of 1.08–1.99, the prevalence of IA rupture was 13% lower for each 0.1-unit increment in AR (odds ratio 0.87, 95% CI 0.80–0.98). Conversely, for every 0.1-unit increase in AR, the prevalence of IA rupture increased by ~ 3% (odds ratio 1.03, 95% CI 1.01–1.06) in the AR range of 3.42–4.08. It is worth mentioning that we observed no association between AR and the prevalence of IA rupture in the AR range of 1.99–3.42.

**Table 3:** Non-linearity addressing using the two-piecewise linear model.

**Fitting by the two-piecewise linear model**	**OR, 95% CI and *P* values**
Inflection point	1.99, 3.42
1.08–1.99	0.87 (0.80, 0.98) <0.0001
1.99–3.42	1.01 (0.98, 1.04) 0.4974
3.42–4.08	1.03 (1.01, 1.06) 0.0020

*Interesting independent variable is AR (per 0.1 change)*.

*Outcome variable is intracranial aneurysm (rupture, non-rupture)*.

*We adjusted for covariates presented in Table 1*.

## Discussion

In this study, we analyzed the relationship of AR with the prevalence of ruptured IA in a large sample set (*N* = 1588), including 663 patients with ruptured IA. The U-shaped relationship was seen here for AR with IA rupture. Interestingly, the non-linearity analysis by the two-piecewise linear model demonstrated here captures more variation than a single-slope logistic regression model: AR appears to follow a different pattern of association with ruptured IA in different ranges.

Previous studies focused only on the “linear” relationship and yielded conflicting results. Twelve studies (sample size ranging from 8 to 425) and two systematic reviews (19 studies, 1553 cases) found a positive correlation between AR and IA rupture (23–36). Eight studies (sample size ranging from 45 to 713) found AR was not associated with ruptured IA (17, 20, 37–42). However, none of these studies defined an applicability range for the associations of AR and ruptured IA (Supplementary Table 1). Indeed, we observed three distinct patterns of AR with ruptured IA in different AR ranges, including negative correlation (AR range 1.08–1.99), no correlation (AR range 1.99–3.42) or positive correlation (AR range 3.42–4.08). This interpretation might offer one explanation as to why the results obtained from previous studies are quite different but seem “reasonable.”

Greving et al. (43) pooled four prospective cohort studies and established PHASES score for prediction of the risk of rupture of incidental intracranial aneurysms. The study confirmed that older, a history of hypertension, current smoker, large-size IA, located in ACA/Pcom/posterior are independent risk factors for aneurysm rupture. This is consistent with our study. However, in this study, we only found that aneurysms located in the MCA are positively associated with the risk of rupture. The previous literature reported that in addition to MCA, there is also ACA/Pcom/posterior. We speculate that this is related to the small number of aneurysms located in ACA, Pcom, and posterior in our study.

We also found that patients with concurrent cerebral microbleeds or irregular IA are associated with a high risk of aneurysm rupture. This is consistent with the previously published literature. Two studies (44, 45) have confirmed that the bleeding rate of CMBs-related rupture is much higher than that in non-CMBs aneurysm patients. In addition, a review also suggested that compared with those with regular aneurysms, aneurysms with irregular shapes have a higher risk of rupture (46).

To investigate how these indicators could influence the differing impact of AR on bleeding risk, we conducted a series of sensitivity analyses. First, when we stratified by age (>60, ≤60), we found that the relationship between AR and IA bleeding risk is still a U-shaped relationship in patients older than 60 years (Supplementary Figure 1). Similarly, in non-smokers, we found a U-shaped relationship between AR and aneurysm rupture, but it was not found in current smokers (Supplementary Figure 2). Therefore, the two sensitivity analyses suggest that the U-shaped relationship between AR and aneurysm rupture in this study seems to be related to the older age and higher percentage of non-smokers of the study population. Our findings are more suitable for non-smokers and elderly patients over 60 years old.

Given that AR is the ratio of the greatest perpendicular height (IA size) to the mean neck diameter, we also evaluated the association between IA size and mean neck diameter and aneurysm rupture, respectively in this study. We observed a positive association between IA size and IA rupture. This is the same as the previous results. These studies found that the unruptured IAs were significantly smaller than the maximum size of ruptured IAs, and the presence of an unruptured IA of ≥7 mm was an independent risk factor for the lifetime occurrence of SAH (43, 47). We also found that the average neck diameter was not associated with bleeding risk. In a prospective large sample study, Mocco et al. (20) found that mean neck diameter was not a statistically significant predictor of rupture. This conclusion is the same as our study.

This research has several strengths of note. First, the sample size of this study is large, ensuring sufficient statistical power. Second, a case-control study design was not used in this study. Compared with previous studies, therefore, the selection bias is reduced. Third, compared to most previous studies that only adjust morphological parameters or demographic characteristics, the adjustment strategy of this study is sufficient and more complete. Fourth, to our best knowledge, it is first to address non-linearity and test interaction on the association of AR with IA rupture. Fifth, sensitivity analysis ensures the robustness of our findings. It roughly explains why the U-shaped relationship between AR and aneurysm rupture can be observed in this study.

Indeed, the interpretation of the findings of this study should be cautious. Several limitations are also noteworthy. First, we only include patients who have clinical symptoms to see a doctor. Therefore, the absence of patients without symptoms will result in selection bias. But such patients are non-ruptured IA, therefore, this would have biased results toward the null. Second, conclusions can be generalized to patients with single IA only, and the correlation of AR and ruptured IA may be different in multiple IA. Third, this study was designed for cross-section, so the causal association between AR and IA rupture could not be confirmed because there is no evidence that the morphological parameters before and after IA rupture are unchanged. Fourth, although we have adjusted for those of measurable confounders, as in all observational studies, there might have been still uncontrolled confounding due to unmeasured differences between IA with and without rupture.

## Conclusion

The independent correlation between AR and ruptured IA is U-shaped. When clinically using AR to estimate the risk of IA bleeding, the different trends within different AR ranges should be judged, especially for the elderly or non-smokers. In addition, older, a history of hypertension, current smoker, large-size IA, located in MCA are independent risk factors for aneurysm rupture. Neurosurgeons can make more detailed and individualized clinical decisions for patients with aneurysms based on these characteristics.

## Data Availability Statement

The raw data supporting the conclusions of this article will be made available by the authors, without undue reservation.

## Ethics Statement

The studies involving human participants were reviewed and approved by Ethics Committee of Zhujiang Hospital. Written informed consent for participation was not required for this study in accordance with the national legislation and the institutional requirements.

## Author Contributions

J-HY wrote the manuscript. S-XS is responsible for data analysis. XZ is responsible for study design and data analysis. Y-MB is responsible for data collection. C-ZD is responsible for the revision of the paper. W-mH and X-LW are responsible for data collection. All authors contributed to the article and approved the submitted version.

## Funding

This work was supported by the National Natural Science Foundation Project (Grant Number: 81974177) and Initiation Plan Project of Clinical Research of Southern Medical University (Grant Number: QD2018N022).

## Conflict of Interest

The authors declare that the research was conducted in the absence of any commercial or financial relationships that could be construed as a potential conflict of interest.

## Publisher's Note

All claims expressed in this article are solely those of the authors and do not necessarily represent those of their affiliated organizations, or those of the publisher, the editors and the reviewers. Any product that may be evaluated in this article, or claim that may be made by its manufacturer, is not guaranteed or endorsed by the publisher.
