# Supplementary figures and images for: U-Shaped Association of Aspect Ratio and Single Intracranial Aneurysm Rupture in Chinese Patients: A Cross-Sectional Study
Source: Front Neurol. 2021 Nov 3;12:731129. doi: 10.3389/fneur.2021.731129 (PMC8598388; doi:10.3389/fneur.2021.731129)

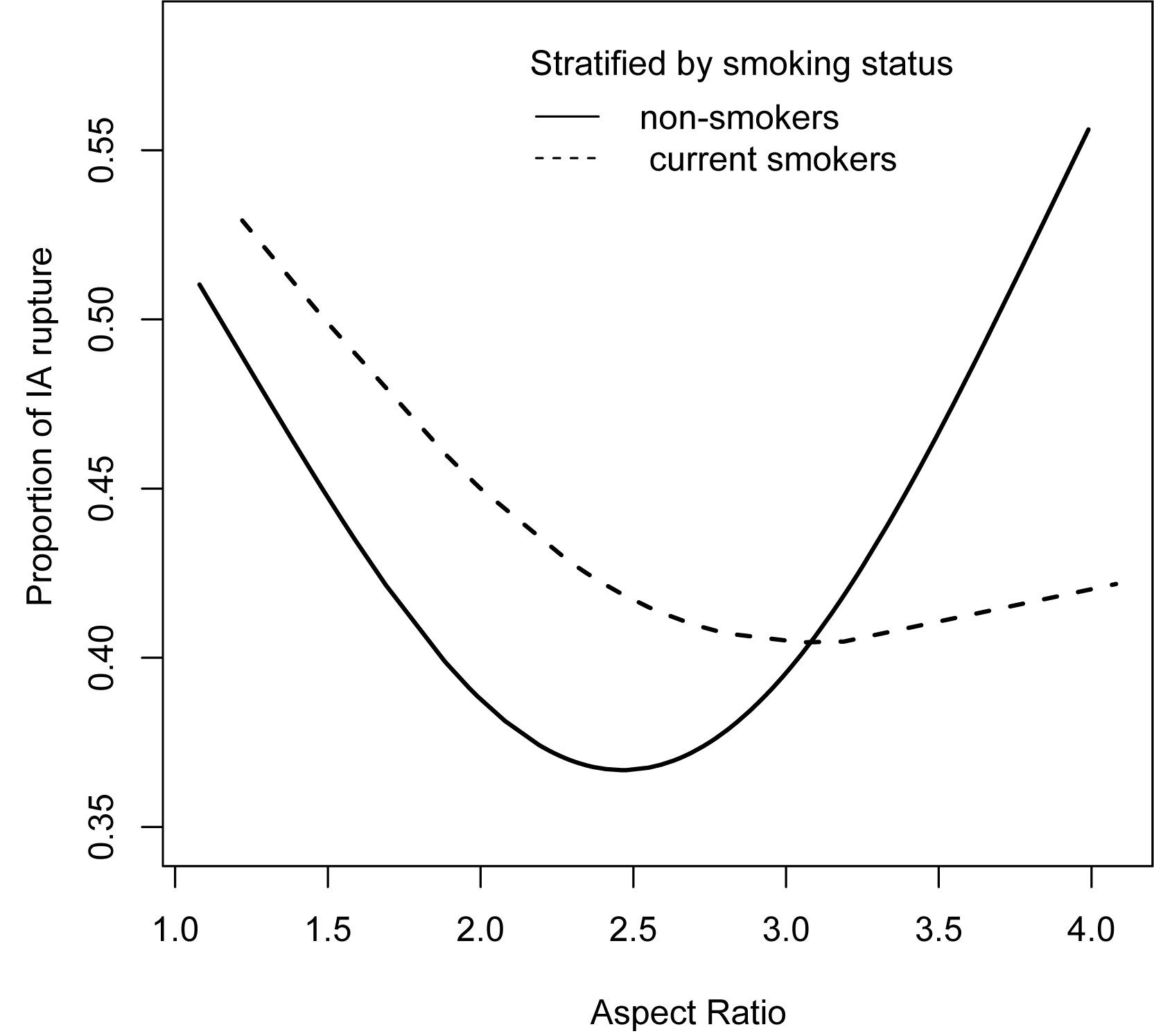

Supplement: Supplementary file 1 [file Image_1.TIF]

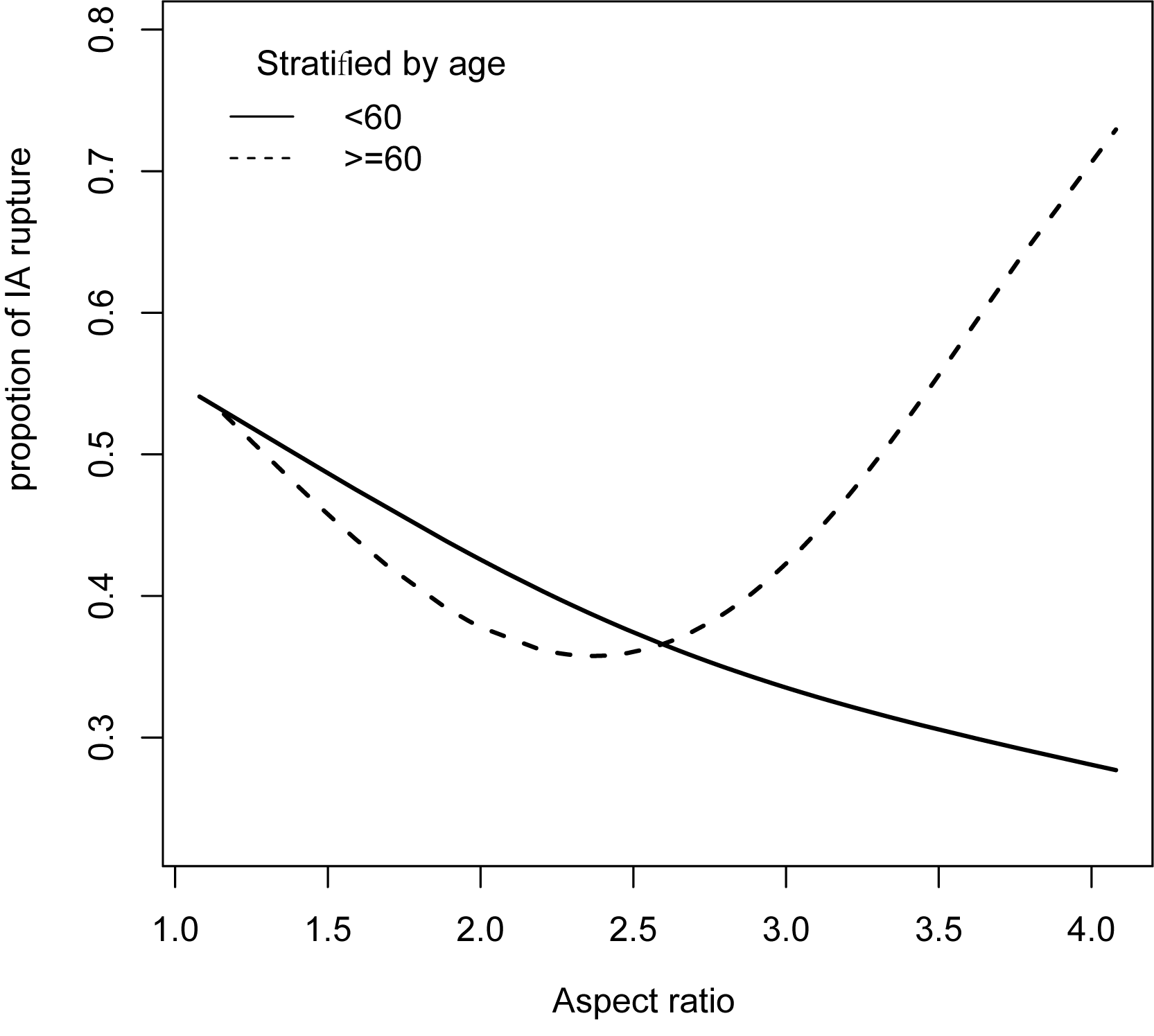

Supplement: Supplementary file 2 [file Image_2.TIF]
