# Supplementary material for: U-Shaped Association of Aspect Ratio and Single Intracranial Aneurysm Rupture in Chinese Patients: A Cross-Sectional Study
Source: Front Neurol. 2021 Nov 3;12:731129. doi: 10.3389/fneur.2021.731129 (PMC8598388; doi:10.3389/fneur.2021.731129)
Supplement: Supplementary file 4 [file Table_1.pdf]

Supplemental table 1: The overview of previous studies which were performed to clarify the relationship between AR to risk of IA rupture

Author(ref)	Year	Study design	No. patients	Study purpose	Involved variables for adjusting or building model	Conclusion	Data analysis	Overview
Jiang, P, et al	2018	Case-control	334	Model-driven	Demography+Comorbidity+Morphological parameters	No correlation between AR and IA rupture	PSM, multivariate logistic regression	Using post-matching cohort to establish model, lack of detailed description of control lead to selection bias. Conditional logistic model should be used.
Lin, N, et al	2013	No description, likely to be a cross-sectional study	89 (IA localized AcoA)	Correlation analysis	Demography+Comorbidity+Morphological parameters	No correlation	multivariate logistic regression	lack of detailed description of study design, some key covariates were not adjusted, nonlinearity and interaction were not considered.
Elsharkawy, A, et al	2013	No description, likely to be a cross-sectional study	1009	Correlation analysis	Morphological parameters only	No correlation	multivariate logistic regression	lack of detailed description of study design, nonlinearity and interaction were not considered.
Fung, C, et al	2019	No description, likely to be a cross-sectional study	1080 (single +multiple IA)	Correlation analysis	Morphological parameters only	No correlation	multivariate logistic regression	lack of detailed description of study design, nonlinearity and interaction were not considered. GEE model was not used for those patients with multiple IA
Mocco, J, et al	2018	Prospective cohort study with nested case-control match	255 patents (unruptured IA)	Correlation analysis	Age, sex, Morphological parameters	No correlation	multivariate logistic regression	nonlinearity and interaction were not considered. Clinical features were not used for constructing fully-adjusted model. However, the evidence power is strongest than other studies
Yasuda, R, et al	2011	Cross-sectional study	155	Model-driven	Morphological	correlation	multivariate logistic	nonlinearity and interaction were not

					parameters only		regression	considered; demography and clinical data were not involved
Tykocki, T, et al	2014	No description, likely to be a cross-sectional study	254	unclear	Morphological parameters only	AR was an risk factor for IA rupture	multivariate logistic regression	lack of detailed description of study design,
Abdurrahim Dusak, et al	2013	No description, likely to be a case-control study	49	Correlation analysis	No variables were adjusted	No correlation	Univariate analysis	Selection of patients maybe lead to selection bias. None of confounders were controlled.
Qiu, T, et al	2017	No description, likely to be a cross-sectional study	429 aneurysms	Correlation analysis	Demography+Comorbidity+Morphological parameters	significant independent factors (positive)	multivariate logistic regression	lack of detailed description of study design, nonlinearity and interaction were not considered.
Feng, X, et al	2017	No description, likely to be a cross-sectional study	548 (618 aneurysms)	Correlation analysis	Demography+Comorbidity+Morphological parameters+ history	No correlation	multivariate logistic regression	lack of detailed description of study design, nonlinearity and interaction were not considered., GEE was not used for multiple IA
You, S H, et al	2010	Case-control matching nested a prospective cohort study	209	Correlation analysis	Demography+Comorbidity+Morphological parameters+ history	AR was an independent risk factor	multivariate logistic regression	nonlinearity and interaction were not considered
Bjorkman, J	2017	cross-sectional study	713 sIAs (multiple)	Correlation analysis	emography+Comorbidity +Morphological parameters+ history	No correlation	Multivariate generalized linear mixed models	Using multivariate generalized linear mixed models to control the internal correlation. nonlinearity and interaction were not considered
Backes, D, et al	2014	No description, likely to be a cross-sectiona study	124 included patients with 302 aneurysms	Correlation analysis	Morphological parameters only	Positively associated with IR rupture	Conditional Multivariate logistic regression	Conditional Multivariate logistic regression maybe incorrect because matching were not used (no description). nonlinearity and interaction were not

								considered
Xavier, M, et al	2015	No description, likely to be a cross-sectiona study	218	Correlation analysis	Demography+Comorbidity+Morphological parameters+ history	No correlation	multivariate logistic regression	nonlinearity and interaction were not considered. The use of ROC confused the correlation analysis and model-based study.
Duan, Z, et al.	2018	No description, likely to be a cross-sectiona study	263 sing, and small IA	Correlation analysis	emography+Comorbidity +Morphological parameters+ history	Positively associated with IA rupture	multivariate logistic regression	nonlinearity and interaction were not considered
Huang, Z Q, et al	2016	No description, likely to be a cross-sectiona study	2674, four centers	Correlation analysis	Morphological parameters only	Positively associated with IA rupture	Conditional Multivariate logistic regression	nonlinearity and interaction were not considered. Conditional Multivariate logistic regression maybe incorrect because matching were not used (no description).
Liang, L, et al	2019	Meta-analysis	46 studies with 2791 IA	Meta-analysis	study type, boundary conditions, solver resolutions, parameter definitions, geometric and hemodynamic parameters used, and results found were recorded	correlate most strongly with IA rupture	-	
Xiang, J, et al	2015	No description	85	Model-driven study for outside validation		Low sensitivity	multivariate logistic regression	
Wang, G X, et al	2018	No description, likely to be a cross-sectiona study	68 patients with mirror PComAAs and aneurysmal subarachnoid	Correlation analysis	Morphological parameters only	Positively associated with IA rupture	multivariate logistic regression	nonlinearity and interaction were not considered. The use of ROC confused the correlation analysis and model-based study.

			hemorrhage					
Jing, L, et al	2015	unclear	69 patients with multiple IA	Unclear	Morphological and Hemodynamic parameters only	Positively associated with IA rupture	AR werethe independently significant rupture factors	nonlinearity and interaction were not considered. The use of ROC confused the correlation analysis and model-based study. GEE model or mix model were not used.
Li M, et al	2013	Self Controlled Case Series	52 with paired IA	unclear	Morphological and Hemodynamic parameters	No correlation	conditional logistic regression analysis	GEE model or mix model were not used. Nonlinearity were not considered
